# A difference that matters: the aortic root anatomy of large animal models vs. humans

**DOI:** 10.3389/fcvm.2025.1699721

**Published:** 2025-12-24

**Authors:** Venessa Strauss, Harish Appa, Paul Human, Ferdinand Vogt, Waled Hadasha, Jacques Scherman, Qonita Said-Hartley, Yvonne Schneeberger, Helga Bergmeister, Lenard Conradi, Bruno K. Podesser, Peter Zilla

**Affiliations:** 1 Strait Access Technologies, University of Cape Town, Cape Town, South Africa; 2 Cardiovascular Research Unit, Cape Heart Centre, University of Cape Town, Cape Town, South Africa; 3 Chris Barnard Division of Cardiothoracic Surgery, University of Cape Town and Groote Schuur Hospital, Cape Town, South Africa; 4 Department of Cardiac Surgery, Artemed Specialist Clinic, Munich, Germany; 5 Department of Cardiac Surgery, Paracelsus Medical University, Nuremberg, Germany; 6 Department of Radiology, University of Cape Town, Cape Town, South Africa; 7 Department of Cardiovascular Surgery, Univ. Hospital Hamburg Eppendorf (UKE), Hamburg, Germany; 8 Center for Biomedical Research and Translational Surgery, Medical University of Vienna, Vienna, Austria; 9 Ludwig Boltzmann Institute for Cardiovascular Research, Medical University of Vienna, Vienna, Austria; 10 Department of Cardiac Surgery, University Hospital of Cologne, Cologne, Germany; 11 Division of Cardiac Surgery, University Hospital St. Pölten, Karl Landsteiner University of Health Sciences, St. Pölten, Austria

**Keywords:** large animal model, anatomical assessment, preclinical, TAVR, aortic root

## Abstract

**Introduction:**

The development of transcatheter aortic valve devices critically depends on preclinical testing in large animal models, yet key anatomical differences between these models and humans remain insufficiently defined. This study evaluated the translational relevance of ovine and porcine models by comparing aortic root anatomy with that of healthy individuals and patients with aortic valve disease.

**Methods:**

Silicone root casts and ECG-gated computed tomography (CT) imaging were used to assess annular, sinus of Valsalva (SOV), and sinotubular junction (STJ) dimensions, as well as coronary ostial height and eccentricity.

**Results:**

Pigs and sheep exhibited significantly lower and more laterally displaced left coronary ostia compared to humans—features that may predispose to coronary obstruction during valve implantation. Body weight correlated with key root dimensions, but wide individual variability precludes its use for selecting individual animals. However, it remains a useful filter for defining cohorts from which suitable subjects can be selected using CT. Sheep demonstrated flatter sinuses and lower STJ heights than pigs and humans, further reducing coronary inflow clearance. In contrast, coronary heights in humans were consistent across valve pathologies, with sinus dimensions being the most variable feature.

**Conclusion:**

Validation of *ex vivo* silicone casting against *in vivo* CT confirmed its suitability for scalable anatomical assessment while aligning with animal welfare principles. These findings support refinement of animal selection strategies and provide an anatomically grounded framework for preclinical evaluation of transcatheter valve technologies.

## Introduction

Validating new transcatheter platforms in large animal models remains a crucial step in translational research (1–3). This process is essential for gaining pivotal insights into product design and performance, ensuring the safety and regulatory approval necessary for subsequent human clinical trials. Despite the widespread success of transcatheter aortic valve replacements (TAVR) over the past 20 years, considerable uncertainty remains about whether the limited anatomical knowledge of the two most used large animal models allows for meaningful inferences regarding clinical risk profiles, particularly in view of high intraoperative pre-clinical mortalities, even in surgical aortic valve replacements (4).

While pigs are the standard model for acute cardiac procedures like percutaneous transluminal coronary angioplasty (PTCA) (5) TAVR present greater challenges due to their requirement for long-term implantation. Consequently, combining acute porcine models with chronic ovine models (6, 7) has evolved as a broadly recognised approach (8). However, this strategy's acceptance is driven more by the absence of better-suited alternatives rather than perfect alignment with clinical reality. Significant disparities between species and clinical implants, including lower coronary ostia, healthy leaflets rather than diseased ones, and varying sizing needs due to increased root compliance, compound the assessment of risks associated with major adverse events such as coronary occlusion or embolisation.

Clinical guidelines, for instance, suggest a minimum coronary height of 10–12 mm for safe TAVR implantation (9, 10). Yet, both pigs (11) and sheep models (12, 13) typically exhibit coronary heights well below this threshold, with precise data being notably sparse. Moreover, there remains considerable uncertainty regarding the eccentricity of coronary ostia within the sinuses of Valsalva (SOV) and their sizes, shapes and orientation. Such anatomical ambiguities significantly limit the predictability of adverse events and the ability to assess their clinical significance.

Furthermore, the frequent absence of pre-implantation imaging in animal models often exacerbates these uncertainties. Unlike in human patients, where valve selection considers precise anatomical dimensions like annular size and coronary height, preclinical research often relies on predictive valve sizes based on non-cardiac measurements (14) such as body weight (11), derived from earlier studies correlating valve size with body size (14). Besides, initial preclinical studies typically test one single valve size, further hampering the assessment of device suitability across diverse anatomical contexts.

Distinguishing between general anatomical limitations of animal models and specific risks associated with devices necessitates pre-procedural imaging and a thorough understanding of the species-specific root anatomy. To better define the latter, we analysed root dimensions using silicone casts and ECG-gated computed tomography (CT) scans in large animal models and compared them against clinically relevant human anatomy.

## Materials and methods

*Ex vivo* analyses of the aortic root of sheep and pigs were conducted using three-dimensional (3D) reconstructions derived from scanned silicone casts. Likewise, 3D reconstructions generated from CT scans were utilised to compare both species' *in vivo* aortic root anatomy with that of humans. CT scans were additionally analysed following clinical conventions (15).

### *Ex vivo* and *in vivo* capture of aortic root anatomy

#### *Ex vivo*: silicone casting

Aortic roots of Merino and Dorper sheep, as well as Landrace pigs and Kolbroek mini-pigs, were sourced from abattoirs following slaughter inherent to commercial meat production for human consumption, independent of study requirements (Table 1).

**Table 1:** Groups for silicone cast roots, based on species and weight brackets.

Silicone Casts (*n* = 66)						
Group #	Species	Breed	Weight brackets (kg)	Source	Age (mo)	*n*
1	Sheep	Merino	35–40 kg	Abattoir CPT	±9	10
2	Sheep	Merino	35–45 kg	Abattoir CPT	±12	10
3	Sheep	Dorper	30–40 kg	Abattoir CPT	±3–4	10
4	Pigs	Landrace	65–80 kg	Abattoir CPT	±4–6	10
5	Pigs	Yorkshire	35–45 kg	VIE	3–4	10
6	Mini Pigs	Kolbroek	40–65 kg	Abattoir CPT	9–12	10
7	Mini Pigs	Aachen	35–40 kg	VIE	9–12	6

Source location: Abattoir CPT, Cape Town, South Africa; VIE, Medical University of Vienna, Austria.

Aortic roots from Yorkshire pigs and Aachen mini pigs were obtained from experimental animals of the Center for Biomedical Research and Translational Surgery, Medical University of Vienna, that had reached the termination point of unrelated approved research projects (Protocol No. GZ 66.009/0068-V/3b/2019, GZ 2020-0.272.252) (Table 1). No animals were sacrificed specifically for heart procurement to produce casts.

Experimental animals were euthanised under deep general anaesthesia induced by intramuscular injection of Medetomidine (0.1 mg/kg) and Ketamine (10 mg/kg), followed by Propofol (1 mg/kg), and maintained with inhaled Sevoflurane (2%–4%) during a rapid intravenous bolus of Potassium Chloride (150 mg/kg) was administered to ensure humane termination per approved ethical protocols.

Hearts were promptly placed in ice-water within 30 min of processing at the abattoir or sacrifice at the experimental facility, then frozen at −6 °C before rigor mortis could occur (16). Before silicone-casting hearts were thawed at room temperature after submersion in water. Hegar dilators were inserted through the left ventricle into the annulus to determine maximum annular diameter, documenting the largest size insertable with reasonable force. Two tin-cured silicone rubbers (AMT Composites; Maitland, Cape Town, South Africa) with shore hardness ratings of 20A and 40A, respectively (each consisting of silicone polymer, crosslinker, and catalyst), were blended in a 1:1 ratio as previously described (17) to achieve the desired shore hardness and flexibility for casts.

**Figure 1:**
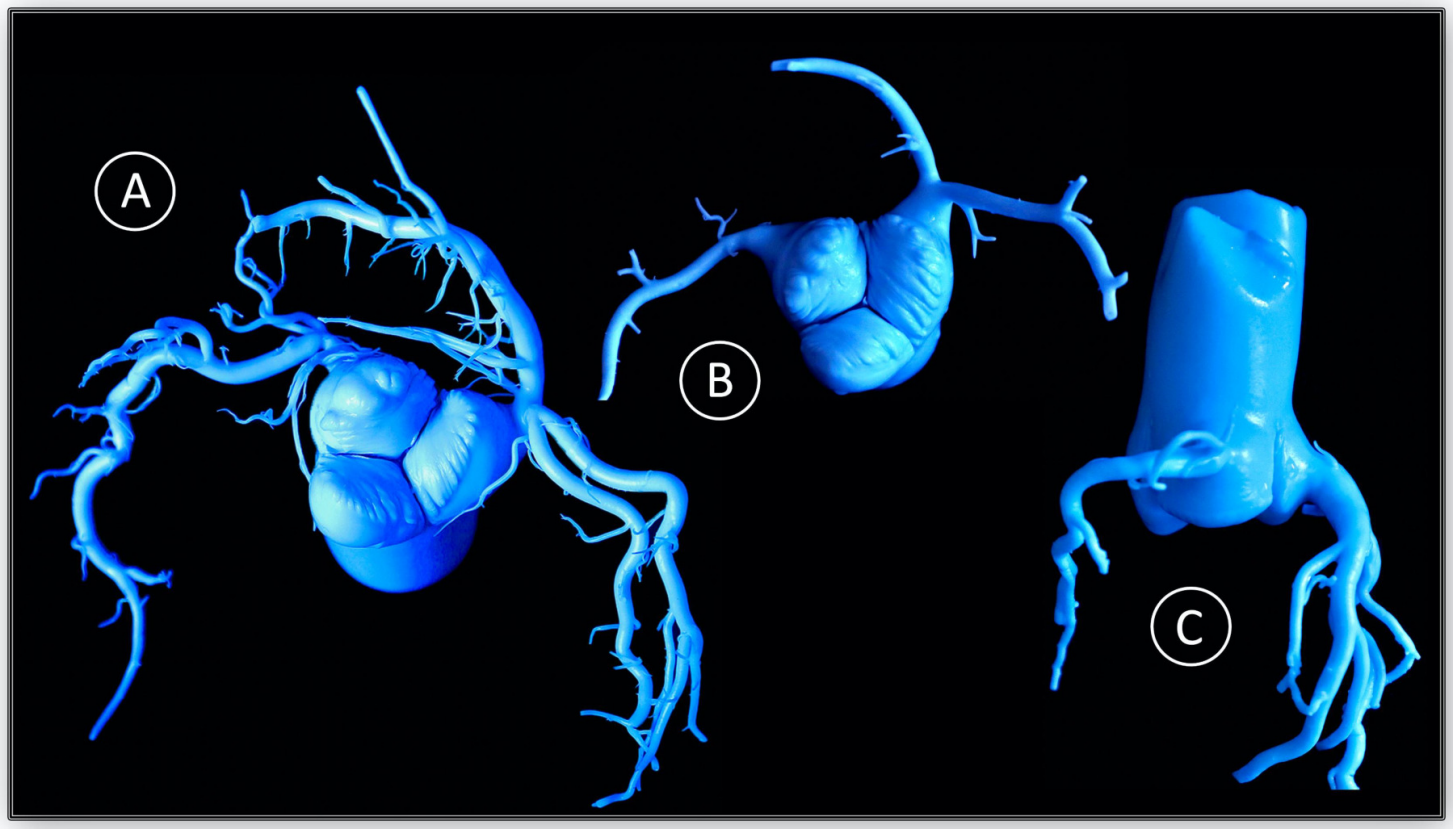
Pressure-controlled silicone-cast of the aortic root of a pig. The leaflet-insertion **(A)**, the eccentricity of the left coronary ostium (LCO) **(B)**, as well as the low height of the LCO **(C)** are discernible.

With the aortic valve closed, silicone was injected through the brachiocephalic artery until all air and water had been displaced, and silicone filled the coronary arteries. The silicone was allowed to cure in the aortic root under diastolic pressure for a minimum of 16 h before the tissue was dissected to allow the *in-toto* extraction of the cast, including the coronary arteries (Figure 1).

To generate scans from silicone casts, a Structured Light Scanner (HP 3D; Pro S3 Palo Alto, CA, USA) was used. Calibration utilised a 30 mm grid consisting of a symmetrical circle grid with black circles of 2 mm diameter evenly spaced 6 mm apart centre to centre, alongside the HP 3D Scan Pro 5.1.0 software. Silicone casts were scanned at maximum resolution. The expected average distance between cloud points and 3D mesh vertex density varied based on sample size, with point distances ranging from 12.1 µm to 20.9 µm, and 3D mesh densities ranging from 3 million to 21.9 million vertices. The 3D models of the aortic root were analysed with Materialise 3-matic medical software (Materialise N.V., Technologielaan 15, 3001 Leuven, Belgium).

#### *In vivo*: ECG-gated computed tomography (CT)

The same acquisition techniques were applied to both animals and humans to obtain CT images throughout the cardiac cycle. CT scans were obtained from animals involved in unrelated research projects approved by the Animal Ethics Committees of the universities of Cape Town (UCT) and Vienna (VIE) (Table 2). Animals were intubated and scanned under anaesthesia to minimise motion artefacts. Following intravenous contrast administration (Ultravist®, Bayer), scans commenced upon reaching an attenuation threshold of 180 Hounsfield units (HU) at 120 kV (Table 2).

**Table 2:** Groups for ECG-gated CT scans including humans. Sheep and pigs were again grouped according to weight brackets.

ECG-gated computed tomography (CT) (*n* = 49)						
Group #	Species	Pathology/breed	Weight/range (kg)	Source	Age	*n*
8	Human	Healthy	79 ± 18	UCT	43 ± 15 years	10
9	Human	AR	71 ± 17	HAM	74 ± 11 years	10
10	Human	AS	66 ± 11	UCT	79 ± 5 years	10
11	Sheep	Merino	20–30	UCT	9 month	8
12	Sheep	Merino	32–52	UCT	12 month	8
13	Sheep	Merino	70–85	UCT	18–24 month	3
13	Pigs	Landrace	44–52	UCT	3–4 month	5
14	Pigs	Yorkshire	36–55	VIE	3–4 month	8

Source location: UCT, University of Cape Town, South Africa; HAM, Hamburg, Germany; VIE, Medical University of Vienna, Austria.

CT images of healthy human hearts were acquired from studies unrelated to heart valve disease (UCT). Images of patients with aortic regurgitation (AR) and aortic stenosis (AS) were obtained from those undergoing evaluation for TAVR (AS: UCT; AR: University Hospital Hamburg Eppendorf; Hamburg, Germany, UKE).

Ethics approval from the University Hospital Hamburg was not required, as deidentified images were supplied (18). Detailed descriptions of ethical approvals at various institutions is available as Supplementary Data.

### Dimensional assessment of aortic root anatomy

**Figure 2:**
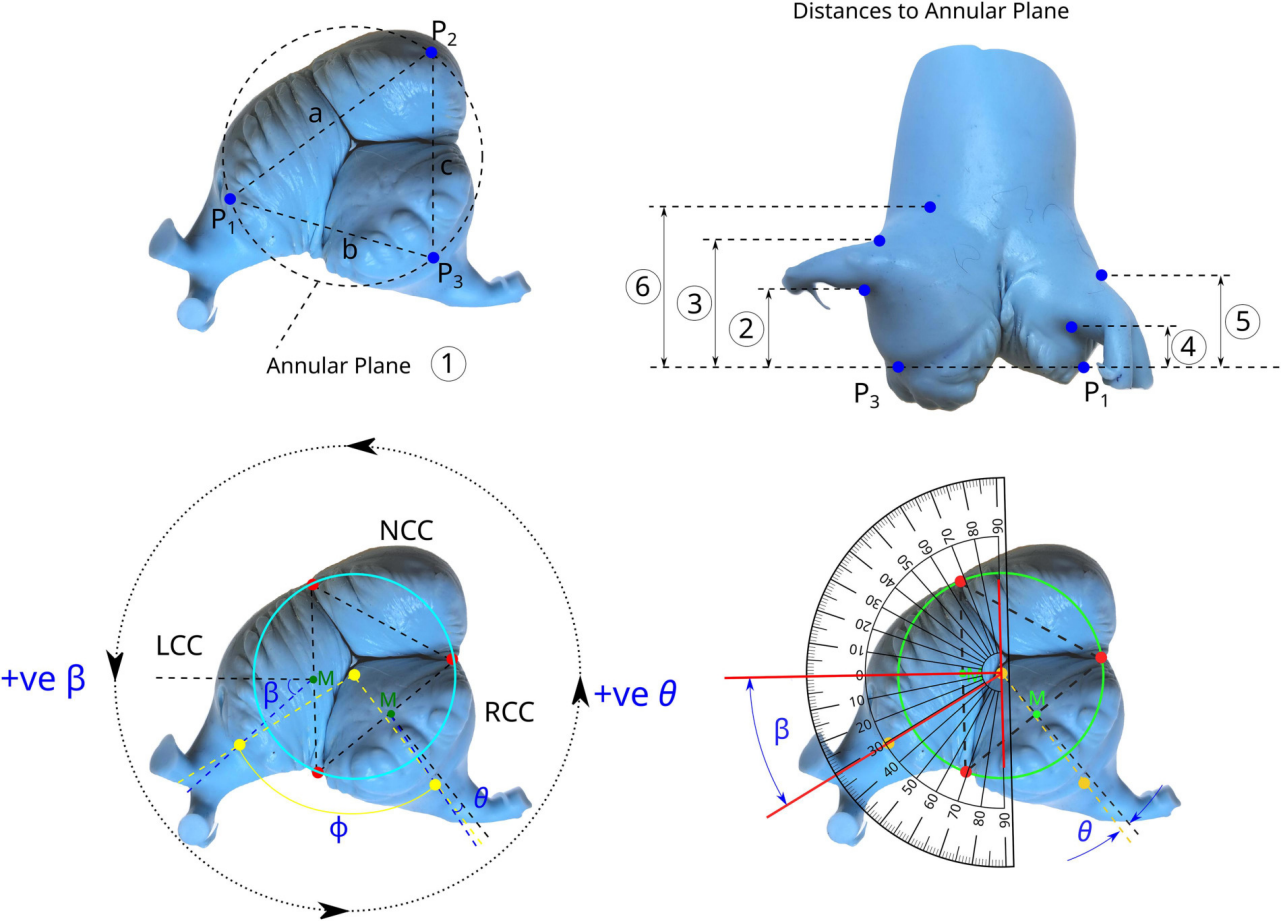
“Geometric” measurements from 3D reconstructions of scanned silicone aortic root casts. Analysis began by pin-marking the nadir of each leaflet insertion to define the annular plane (top left), followed by vertical measurements (top right) to: the lowest (②) and highest (③) point of the RCO; the lowest (④) and highest (⑤) point of the LCO; the STJ, measured from P3 to the post-sinus level of the RCC (⑥). Coronary ostial eccentricity was assessed by comparing a radial line through the midpoint between adjacent commissures with a line through the midpoint of the LCO (β) and RCO (θ). The intercoronary angle (φ) between the midpoints of the coronary ostia was also measured (bottom left and right).

**Figure 3:**
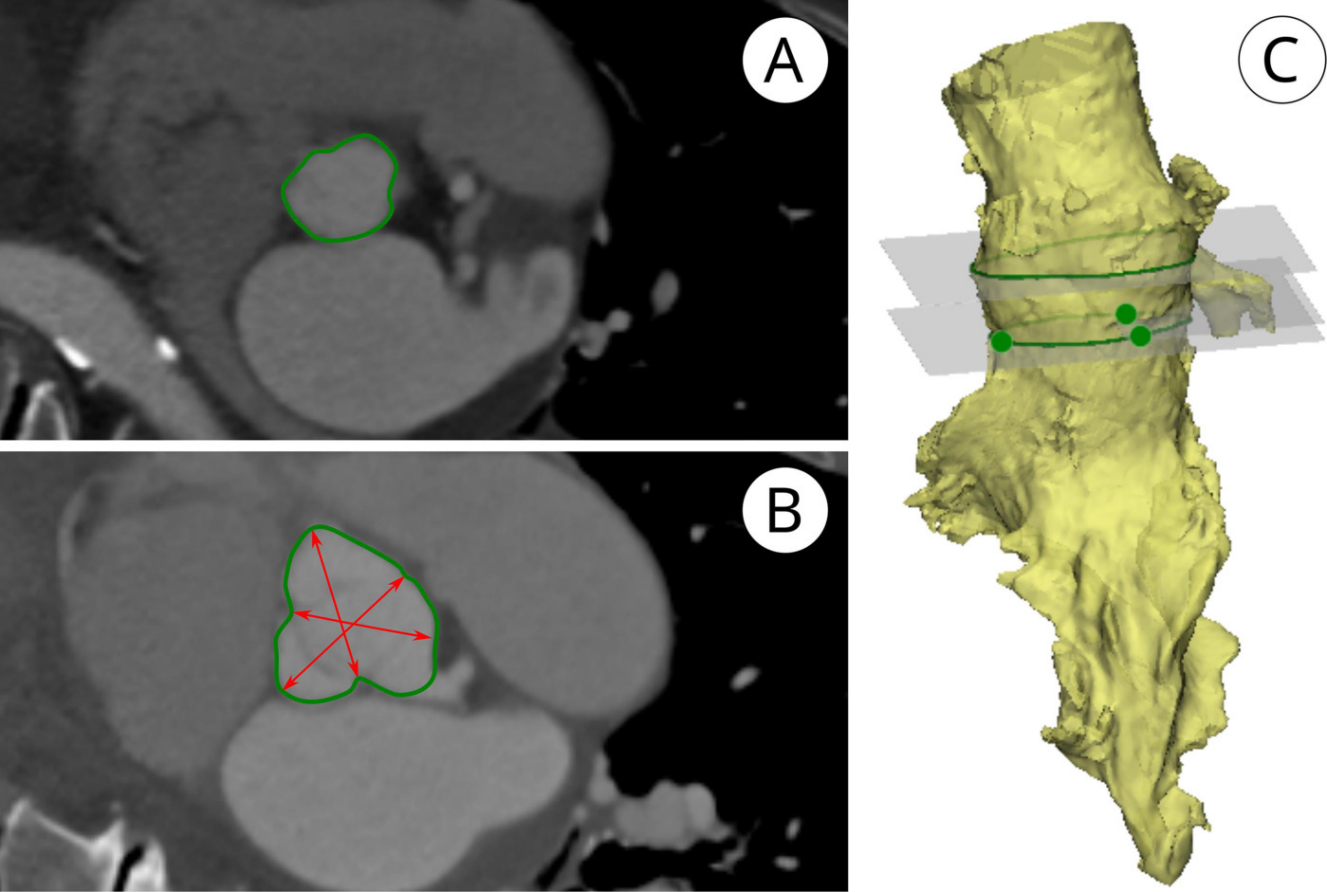
2D CT-based “clinical” assessment of aortic root dimensions. **(A)** Annular diameters were derived from the annular circumference. **(B)** illustrates SOV “bulging,” measured as the linear distance from the midpoint of the sinus at its widest level to the opposing commissure. **(C)** 3D reconstructions of a CT scan.

The 3D models of aortic roots were extracted from CTs as well as structured light scans of silicone casts using Materialise Mimics 25.0 (Materialise N.V. Technologielaan 15, 3001 Leuven, Belgium) and analysed with Materialise 3-matic (Materialise N.V. Technologielaan 15, 3001 Leuven, Belgium). The analysis of scans of silicone casts was only based on external cast landmarks (Figure 2), whereas CT analyses also included intraluminal measurements (Figures 3A–D) per established clinical conventions (15).

Reflecting the 30%–40% phase of the cardiac cycle both the *ex vivo* root cast groups and the *in vivo* CT groups determined five primary dimensional categories: (1) **Diameters** [(a) Annulus (Ann), (b) STJ (c) SOV; (2) **Heights** [(a) Annulus to STJ, (b) Annulus to lowest point of the LCO and RCO, (3) **Angle of deviation** of coronary ostia from the sinus midline (“eccentricity”) and **Ostial diameters and ellipticity** and (4) **Dimensional ratios** [(a) Annulus to SOV (Ann:SOV) and (b) Annulus to STJ (Ann:STJ). To assess the area and circularity of the coronary ostia, the longest (horizontal) and shortest (vertical) diameters were measured. The surface area of the elliptical ostium was calculated using the formula *A* *=* π*ab*, where *a r*epresents half of the longest diameter and *b* represents half of the shortest diameter. The circularity of the ostium was quantified as the ratio of the shortest to the longest diameter.

#### Root dimensions derived from scanned silicone casts

Anatomical landmarks were identified on 3D reconstructions of the scanned silicone casts. As a first step, the annular plane was defined by aligning the nadirs of all three cusps. Subsequently, planes at the level of the SOV and the STJ were established at the widest point of the sinuses and the narrowest post-sinus level, respectively *(“3D Geometric Method”)*. Landmark points on these planes were intersected with virtual circles to define the diameters of the annulus, SOV, and STJ. Coronary heights were measured as the distance from the annular plane to the lowest point of each coronary ostium. Axial projections were used to determine the midline deviation of the coronary ostia relative to the SOV. Ostial diameters were calculated as the mean of horizontal and vertical diameters. Ellipticity related to horizontal and vertical maximum diameters (Figure 2).

#### Root dimensions obtained from CT scans

CT-derived dimensions were obtained using the clinically established method based on luminal anatomical landmarks. Annular diameters were calculated from the perimeter to account for non-circular geometries (15). The SOV was measured as the mean distance from the widest mid-point of each sinus to the opposing commissure *(“2D CT-Distance Method”)* (15). Coronary heights were referenced to the annular plane defined by the nadirs of the valve leaflets. To enable direct comparison with the measurements obtained from the silicone casts, dimensional analyses were additionally performed on 3D reconstructions of all CT scans using the same method as applied to the silicone casts (Figure 3).

### Statistical analysis

Inferential statistical analyses of independent variables, both measured and derived (e.g., Ann:STJ), were performed using JMP Pro (version 18.0.1, SAS Institute Inc., Cary, NC, USA). In some cases, partition modelling, without validation due to limited sample size, was used to identify cut points for continuous numerical independent variables. Principal dependent variables were defined as the degree of coronary rotation, coronary height and Ann:SOV. However, Ann:SOV and its components were interchangeably used as independent variables.

Normality of distribution of continuous numerical variables was evaluated using the Shapiro–Wilk test with either parametric or non-parametric tests subsequently used for normally or skewed independent variables, respectively. This involved either Analysis of Variance testing, with *post hoc* comparisons performed using the Tukey–Kramer HSD/Student's *t*-tests in the case of the former, or Kruskal–Wallis/Wilcoxon tests, with *post hoc* Steel-Dwass test used in the case of the latter. Both *post hoc* tests permitted multiple comparisons without correction.

The degree of congruency between manually obtained and image-analytical continuous numerical data was assessed by linear regression using Pearson's correlation coefficient.

Continuous variables were reported as means ± standard deviation throughout. Statistical significance was assumed for two-sided *p*-values when *p* < 0.05.

## Results

Compared with humans, both pigs and sheep differed most distinctly in their height of the LCO (4.2 times and 3.3 times lower in pigs and sheep than in humans) and its distinct deviation from the midline of the sinus towards the left/right commissure.

### *Ex Vivo* dimensions (silicone root casts)

Supplementary Table S3 is available as supplementary Data and contains the full dataset used for analysis.

Overall, annulus dimensions derived from silicone casts were significantly smaller compared to weight- and breed-matched CT-derived measurements, by 11% in sheep (*p* < 0.02) and 16% in pigs (*p* < 0.0001). This trend was confirmed for SOV (*p* < 0.0001 in sheep and pigs) and STJ height (*p* < 0.01 in sheep and pigs). Passive annular dilation using Hegar dilators at moderate insertion force resulted in a non-significant mean increase in diameter of 11.3% ± 8.5% in sheep and 9.6% ± 10.9% in pigs.

The coronary height was significantly higher in pigs than in sheep (LCA: 4.3 ± 2.1 mm vs. 3.2 ± 1.0 mm; *p* < 0.01; RCA: 9.2 ± 2.9 mm vs. 6.4 ± 1.4 mm *p* < 0.0001). The RCO diameter was significantly bigger in pigs than sheep [4.9 ± 1.8 mm vs. 3.9 ± 1.0 mm; *p* = 0.01] while the LCO diameter was similar [6.9 ± 2.0 mm vs. 7.2 ± 1.0 mm; *p* = 0.53]. The horizontal ostial ellipticity of both ostia [LCO 0.83 ± 0.11 vs. 0.82 ± 0.07; *p* = 0.95; RCO 0.75 ± 0.10 vs. 0.75 ± 0.13; *p* = 0.81] was similar in pigs and sheep, respectively.

No significant interspecies differences were observed in SOV bulging, as measured by the Ann:SOV ratio (0.87 ± 0.06 in pigs vs. 0.85 ± 0.06 in sheep; *p* = 0.19), or in the midline deviation of coronary ostia. The midline deviation of the LCO towards the left/right commissure was 24.3 ± 7.5° (8.6–42.7°) vs. 26.3 ± 5.9°(11.3–37.8°) in pigs and sheep respectively (*p* = 0.26), and for the RCO 6.2 ± 6.8° (0.1–33.6°) vs. 6.7 ± 5.2° (0.2–19.5°) towards the right/left commissure, in pigs and sheep respectively (*p* = 0.73).

In sheep, neither breed (weight-matched Merino vs. Dorper) nor age (3, 9, and 12 months, weight-matched) significantly influenced annulus size or coronary height. However, partition modelling identified a modest but significant correlation between body weight and annulus size, both across breeds (<37 kg: 20.8 ± 1.5 mm vs. > 37 kg: 22.5 ± 1.9 mm; *p* = 0.007) and within breeds (adult Merino sheep, 9–12 months: <37 kg: 20.9 ± 1.4 mm vs. >53 kg: 22.9 ± 1.4 mm; *p* = 0.0027). The annulus diameter showed a strong correlation with SOV size (*P* = 0.0005) and a mild correlation with LCO height (*p* = 0.015). Partitioning revealed an annulus diameter threshold of >21 mm to be associated with a significantly greater LCO height (3.5 ± 0.9 mm vs. 2.7 ± 0.83 mm, *p* = 0.013), and a threshold of >22.6 mm for increased RCO height (7.4 ± 1.1 mm vs. 5.9 ± 1.2 mm; *p* = 0.003).

**Figure 4:**
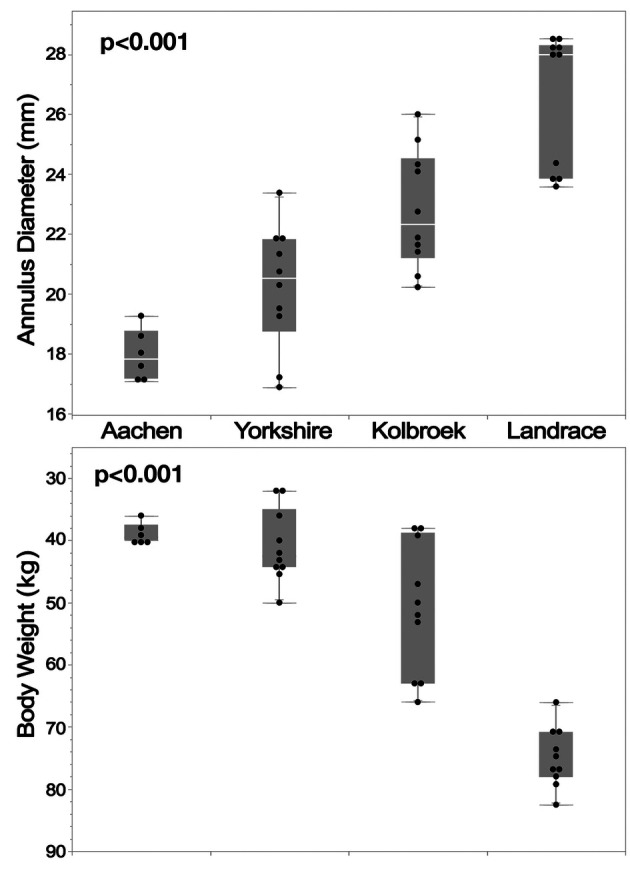
Silicone cast-based comparison across different pig breeds shows a clear correlation between body weight and annulus diameter. This suggests that annulus size is primarily determined by weight rather than breed-specific characteristics.

**Figure 5:**
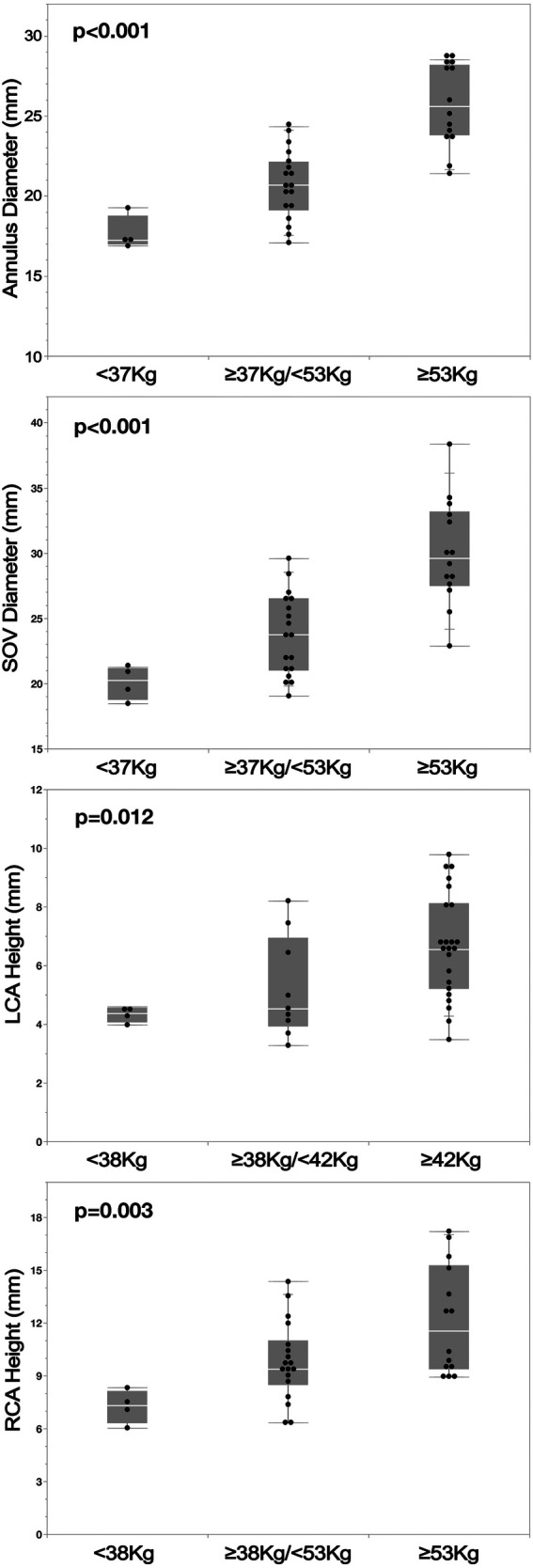
(Casts) Partition modelling in pigs demonstrated a weight-dependent correlation across all four measured parameters: annulus diameter, SOV diameter, and the heights of the LCO and RCO. The correlation was least pronounced for the height of the LCO.

In pigs, partition modelling also demonstrated significant correlations between weight and annulus diameter, SOV dimensions, and coronary height (Figures 4, 5). Annulus size increased progressively with weight: 19.5 ± 2.6 mm (<43 kg) vs. 21.8 ± 1.7 mm (≥43 kg to <66 kg) vs. 26.4 ± 2.2 mm (≥66 kg); *p* < 0.001. Since breed categories coincided with weight brackets, this correlation remained significant across breeds (*p* < 0.0001; Figure 4). Larger annular diameters were associated with progressively higher coronary heights [LCO height: <19.5 mm: 2.0 ± 0.7 mm (1.3–3.5 mm); ≥19.5 mm: 5.0 ± 1.8 mm (2.3–8.0 mm); *p* < 0.0001; RCO height: <20.6 mm: 6.8 ± 1.4 mm (4.9–8.9 mm); 20.6–25.2 mm: 9.5 ± 2.2 mm (6.9–14.3 mm); ≥25.2 mm: 12.2 ± 3.1 mm (7.3–16.2 mm); *p* = 0.0004.

### *In vivo* dimensions (CT)

Supplementary Table S4 is available as supplementary Data and contains the full dataset used for analysis.

CT analyses enabled the comparison of aortic root dimensions between sheep, pigs, and healthy humans, as well as between human patients with AR or AS and healthy human controls.

Perimeter-derived root diameters obtained from clinical CT measurements did not differ significantly from those calculated using circles intersecting the leaflet nadirs in 3D reconstructions (Supplementary Table S4), except for the SOV, where measurements by sinus-to-commissure distance were significantly smaller than the diameter of a circle encompassing all three sinuses. Therefore, except for patients with AR, all Annulus-to-SOV (Ann:SOV) ratios derived from clinically established CT analyses exceeded 0.80. Related to healthy humans (Ann:SOV = 0.82 ± 0.04), the “bulging” of the SOV was mildly less pronounced in pigs (Ann:SOV = 0.87 ± 0.05; *p* = 0.1) but significantly flatter in sheep (0.91 ± 0.06; *p* < 0.001). In contrast, SOV measurements based on circular diameters in 3D reconstructions yielded lower Ann:SOV ratios with humans and pigs being similar (0.73 ± 0.06 and 0.73 ± 0.05, *p* = 0.96), and sheep again being highest (=least bulging) (0.81 ± 0.07; *p* = 0.06).

The STJ height was also significantly lower in sheep (17.6 mm) compared to pigs (20.8 mm; *p* < 0.0004) and humans (21.3 mm; *p* < 0.0001). Among sheep, Merinos raised on sparse African pastures (30.2 ± 8.6 kg) had smaller annular diameters (25.6 ± 1.3 mm vs. 32.6 ± 3.1 mm; *p* = 0.01) and lower, though not significantly different, LCO heights (2.4 ± 1.3 mm vs. 3.6 ± 0.6 mm) compared to their 2.7-times heavier stall-fed counterparts (81.3 ± 5.5 kg).

**Figure 6:**
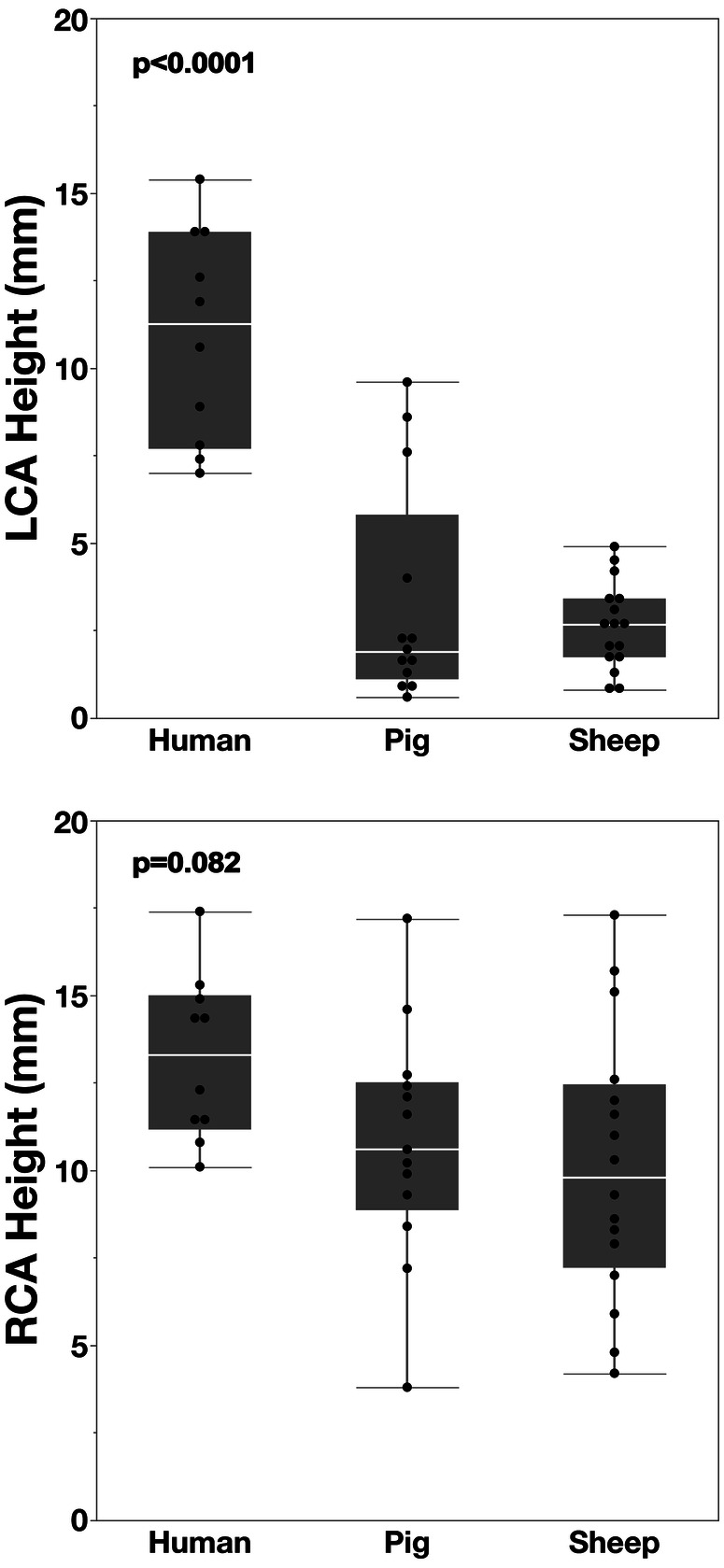
Comparison of CT-derived coronary ostial heights across species. In healthy humans, the LCO height was 4.2 times greater than in adult sheep and 3.3 times greater than in growing pigs. By contrast, the RCO height was similar among humans, pigs, and sheep.

The most distinct difference between adult sheep (39.8 ± 22.1 kg), growing pigs (52.1 ± 9.1 kg) and healthy humans was the LCO height (2.6 ± 1.2 mm; 3.3 ± 3.2 mm and 10.9 ± 3.0 mm, respectively *p* < 0.0001). In contrast, the RCO height varied within a much narrower and statistically non-significant range (10.1 ± 3.9 mm; 10.8 ± 3.4 mm vs. 13.2 ± 2.4 mm; *p* = 0.07) (Figure 6).

The median LCO diameter in healthy humans (7.7 ± 2.7 mm; 5.3–12.4 mm) was smaller than in pigs (12.2 ± 2.6 mm; 7.6–17.6 mm; *p* = 0.007) and sheep (11.7 ± 1.8 mm; 9.0–15.5 mm; *p* = 0.007). Similarly, the median RCO diameter was the biggest in pigs (6.9 ± 1.6 mm; 5.4–11.7 mm) as opposed to humans (5.3 ± 1.6 mm; 3.4–8.3 mm; *p* = 0.029) and sheep (5.5 ± 1.8 mm; 2.6–9.1 mm; *p* = 0.97).

**Figure 7:**
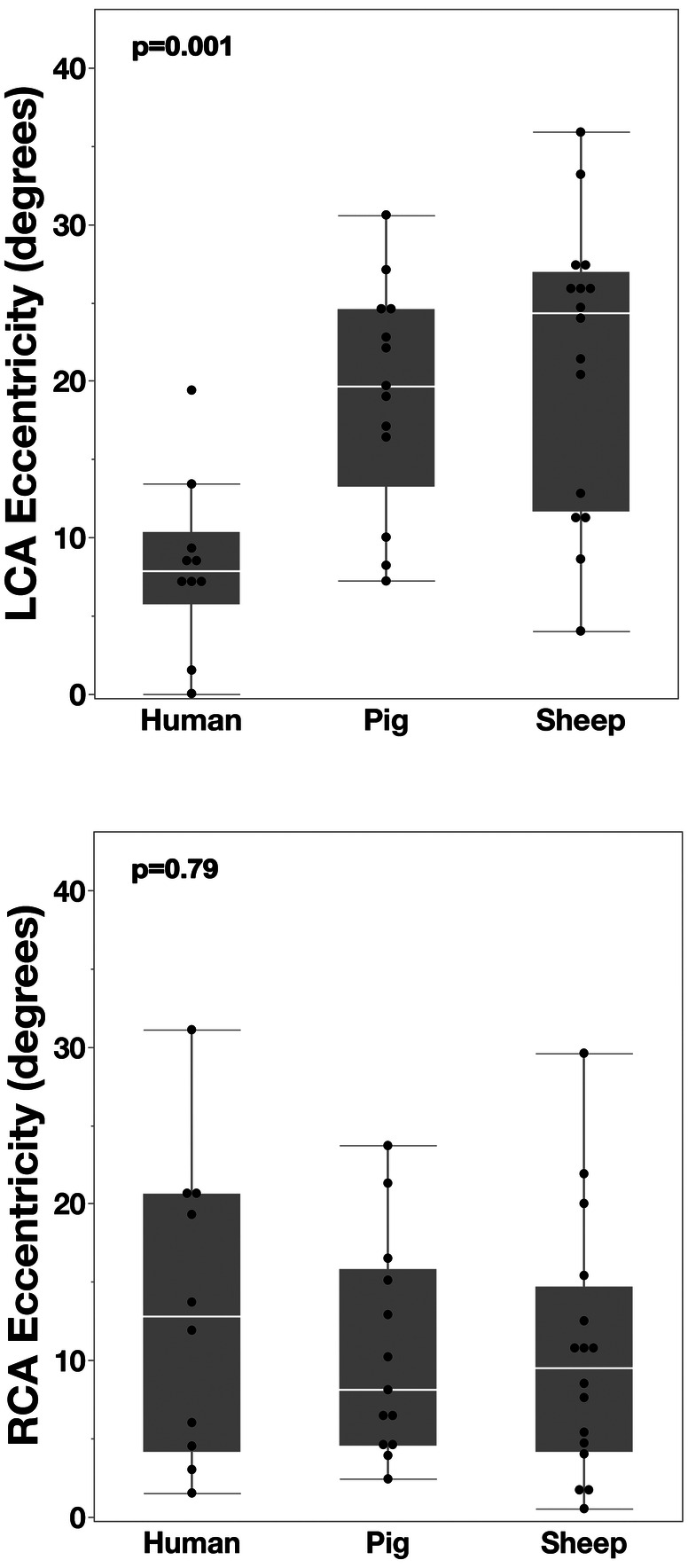
*Eccentricity of CT-derived coronary ostia relative to the sinus midline.* In healthy humans, the LCO is positioned near the midline, whereas in both pigs and sheep, it is distinctly shifted towards the left/right commissure. By contrast, the RCO shows a mild and consistent shift towards the right/non-coronary commissure across all three species.

Coronary eccentricity from the sinus-midline was modest across all three species for the RCO (ranging from 10.3 ± 8.1° to 13.2 ± 9.7° towards the R/NC commissure; NS), but the LCO was significantly more central in humans (8.2 ±5.5° towards the L/R commissure) compared to pigs (19.1 ± 7.3°; *p* = 0.007) and sheep (21.2 ± 9.1°; *p* = 0.003) (Figure 7).

**Figure 8:**
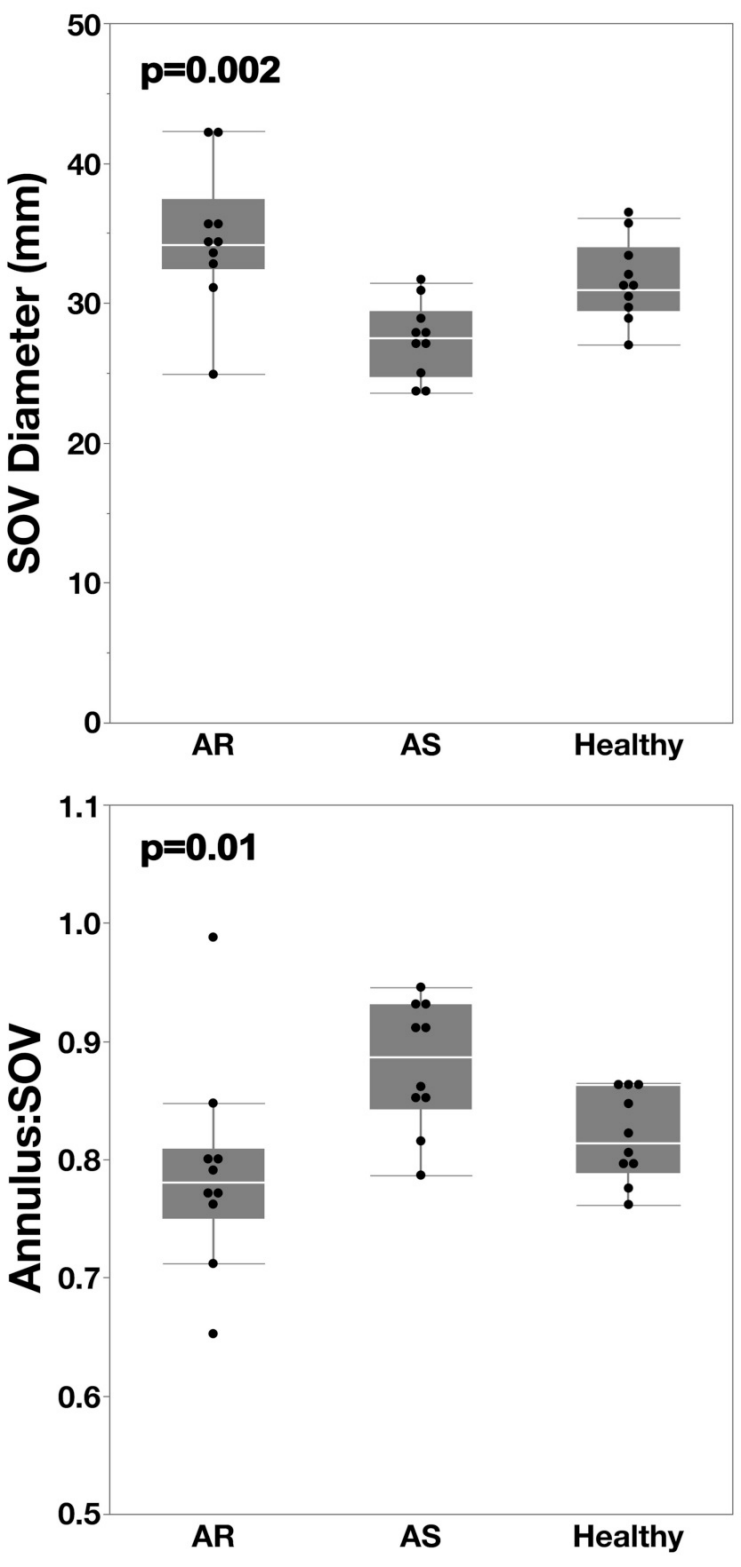
CT-derived ratio of annulus diameter to SOV diameter using conventional clinical 2D CT-measurements. The SOV diameter was determined by measuring the distance from the deepest mid-point of each sinus to the opposing commissure., Patients with AS exhibited a significantly smaller SOV, resulting in the highest annulus-to-SOV (Ann:SOV) ratio among the groups.

In humans, valve pathology had no significant influence on coronary height, coronary ostial “eccentricity” from the sinus midline, or STJ height. The most pronounced difference was a smaller SOV diameter in AS (27.3 ± 2.7 mm) compared to healthy individuals (31.4 ± 2.9 mm; *p* = 0.03), while it was modestly bigger in patients with AR (34.6 ± 5.1 mm; *p* = 0.23) (Figure 8). Using circular SOV measurements from 3D reconstructions, this difference also remained significant between healthy and AS patients (*p* = 0.045).

## Discussion

The primary intention of the study was to generate robust anatomical data on the aortic root in the two most widely used large-animal models—pigs and sheep—alongside human aortic root anatomy, both normal and pathological, to enable clinically pertinent interspecies comparison. It aimed primarily at the translational relevance of preclinical testing and to better inform the design and safety of future transcatheter procedures.

As catheter-based therapies evolve to address increasingly complex interventions, there is a renewed need to recognise the intricate structural relationships of the aortic root. Early transcatheter development primarily targeted valve replacement for calcific AS—a relatively well-characterised pathology with clearly defined anatomical landmarks. Since then, the scope of endovascular treatment has broadened considerably. Contemporary approaches now include coronary re-access after TAVR, devices for non-calcific valve disease, reinterventions, and endografts designed to also address the pathology of the ascending aorta.

Whereas previous anatomical studies of the aortic root in large-animal models typically focused on a single species, breed, geographic region, or husbandry condition (11, 19, 20). Our study aimed to reflect the practical realities of device development. In this setting, sheep and pigs are often used complementarily. Breed selection is largely determined by local availability, while husbandry practices—such as stall feeding vs. pasture grazing—depend on whether short- or long-term follow-up is required.

As a result, the dataset derived from 128 aortic roots across pigs, sheep, and humans was inherently heterogeneous. The resulting diversity introduced greater statistical complexity, which was partly resolved through partition modelling. This method enabled the evaluation of key variables—including breed, age, and weight in animals—and facilitated interspecies comparisons encompassing both healthy and diseased human valves with stenotic or regurgitant lesions.

Our focus on post-juvenile anatomy marks a shift from the earlier emphasis on age-related bioprosthetic calcification (21–24) toward dimensional factors now central to transcatheter device development—namely, deliverability, anchoring stability, and coronary access. In practice, however, most large-animal studies over the past 25 years have already used sheep aged 5–10 months (25–28). These were often combined with subcutaneous rat models to address the still unresolved issue of premature calcific leaflet degeneration, while also acknowledging the minimal calcification typically seen in juvenile sheep (29).

Historically, CT data have remained scarce due to ethical constraints and resource limitations, hindering cross-species anatomical comparisons. Our validation of *ex vivo* silicone casts against *in vivo* CT scans demonstrated that species-specific anatomy can be established without subjecting large numbers of animals to the additional stress of CT imaging. This not only addresses ethical concerns related to animal welfare but also aligns with the 3Rs principles—replacement, reduction, and refinement (30). By enabling anatomical assessment without repeated or invasive procedures, this approach facilitates the generation of more robust datasets. We did, however, observe a mild but consistent reduction in dimensions before casting, suggesting that these changes are more likely attributable to post-mortem changes than to the casting process itself. Accordingly, the primary value of silicone casts lies in enabling large-scale analysis of anatomical relationships, while ECG-gated CT will likely remain the gold standard for accurately measuring absolute individual dimensions.

It is striking that the few available CT data originate from pig studies (11), even though the vast majority of preclinical heart valve research has been conducted in sheep. While pigs more closely resemble human cardiac anatomy, for example, in their right-dominant coronary circulation (31)—sheep have historically been preferred due to moderate somatic growth, broad availability, and ease of husbandry (6, 7). Addressing this disconnect between existing anatomical data and the predominant preclinical model, we included both pigs and sheep in our study to enable an interspecies comparison that reflects the reality of contemporary research and development.

Before comparing anatomical differences between species, we addressed a fundamental methodological issue in preclinical research: the common practice of estimating annulus size and coronary height based on body weight or age. These parameters are routinely used to select animals for experimental studies, relying on extrapolation from prior experience rather than on screening CT scans (11)—largely because CT imaging is often unavailable in standard animal research settings.

Partition modelling in both sheep and pigs revealed a significant correlation between body weight and annulus size, as well as with LCO height and SOV diameter, independent of breed or age. While age often serves as a proxy for weight gain, we showed that it is not an independent predictor in its own right. Within the same Merino breed, sheep raised on concentrated stall feeding and weighing nearly three times more than their older counterparts raised on sparse African pastures had distinctly larger annuli. While their LCO heights were also greater, the difference did not reach statistical significance. These findings further support the conclusion that root anatomy is influenced primarily by body mass, not chronological age.

In pigs, the correlation between weight and annulus diameter was even more pronounced and extended consistently to SOV and coronary height, maintaining statistical significance across breeds, including those commonly referred to as “mini pigs”. Among the few studies reporting CT-based measurements, Lupinski et al. (11) confirmed the relationship between body weight and annulus diameter in pigs. However, they did not observe the association we found between annulus diameter and LCA height. This discrepancy may stem from methodological differences, particularly our use of partition modelling, which may have better captured non-linear or interaction effects. Notably, similar correlations between annulus size and both body height and surface area have also been described in humans (14) underscoring the broader relevance of body size over age in determining root dimensions.

However, the wide confidence intervals we observed—despite statistically significant correlations—indicate that even optimal weight brackets do not permit reliable prediction of individual anatomical dimensions. Nonetheless, these correlations are valuable in guiding the selection of the most appropriate weight bracket for pre-screening, thereby improving the likelihood of identifying anatomically suitable candidates. This highlights the limitations of relying solely on weight-based selection while reinforcing the importance of pre-procedural imaging in studies that depend on precise anatomical matching, for device sizing, implantation strategies, and reliable interpretation of outcomes.

In our species comparison, we first identified anatomical features in which both pigs and sheep differed significantly from humans. The most pronounced and potentially consequential of these was the position of the LCO. In both animal species, the LCO was located three to four times lower than in humans and exhibited greater lateral deviation toward the left/right commissure. In contrast, the RCO—generally higher in all species—was positioned near the sinus midline and showed only minimal variation in height.

**Figure 9:**
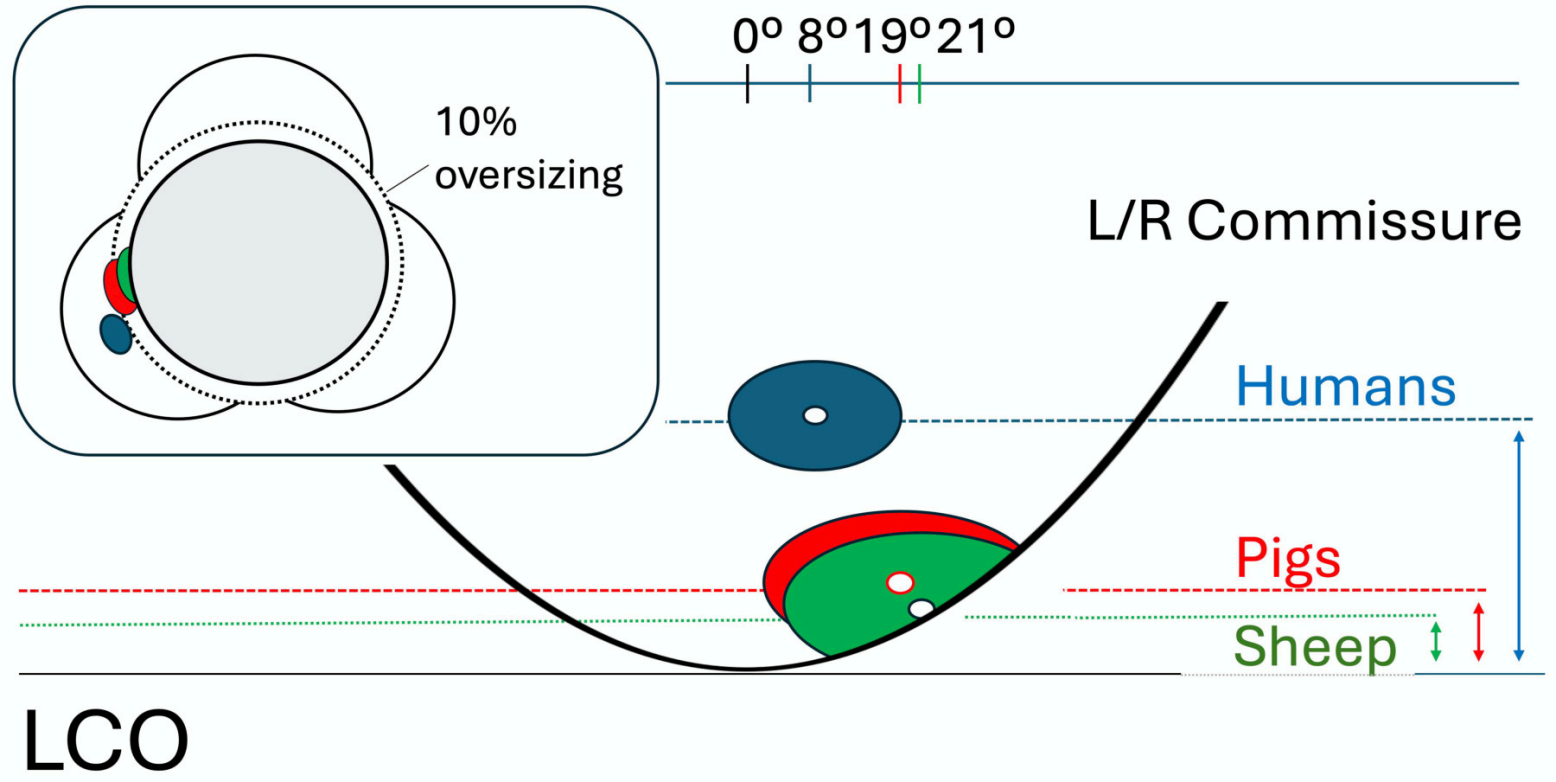
Schematic representation of the left SOV and the insertion line of the left aortic valve cusp. The figure illustrates that the already low-lying LCO of pigs and sheep lies even closer to the hinge line of the leaflet than its vertical height from the nadir alone would suggest, due to its eccentric position relative to the midline. The inset demonstrates how, in sheep, the LCO may becomes obstructed when a device such as a TAVR is implanted with only 10% oversizing. The larger coronary ostia can compensate for this only to a very limited extent.

For the LCO, the combination of reduced height and a clockwise shift toward the commissure places it even closer to the leaflet insertion line. As shown in Figure 9, an LCO that is three to four times lower and abuts the complex, scalloped curve of the leaflet hinge sits nearer to the narrowest part of the sinus than its vertical height from the leaflet nadir alone would suggest. In sheep, even the relatively modest oversizing recommended for balloon-expandable TAVR is therefore predicted to occlude the coronary orifice in most cases. This critical anatomical constraint is partially offset by the more funnel-shaped morphology of the ostium in both sheep and pigs. With diameters roughly twice those in humans and an inflow zone about 3.5 times larger, some coronary perfusion may still occur in borderline cases. Nonetheless, CT-based preselection of animals should strongly consider not only absolute coronary height but also the extent of deviation from the sinus midline toward the commissure to allow a better distinction of species-specific coronary occlusions from this most feared but rare occurrence in patients (9, 10).

Beyond their shared differences from humans, important distinctions between sheep and pigs themselves may critically influence their suitability for transcatheter therapies. Some of these differences are well known. Notably, sheep lack a membranous septum for the conduction system (32) and their valve leaflets are particularly thin and fragile, positioned 1–2 mm lower than in both humans and pigs (33). Most importantly, sheep exhibit a left-dominant coronary circulation without preformed collaterals (19), in contrast to the right-dominant system seen in pigs and humans (20). In contrast, the most consequential anatomical differences—those involving the SOV and the STJ—have received surprisingly little attention to date.

The SOV provide a critical diastolic inflow space for the coronary arteries, particularly when potentially obstructive device frames are present. As such, the annulus-to-sinus ratio—reflecting the degree of sinus bulging—is a key parameter for assessing procedural safety. Two methods are commonly used to measure sinus bulging, potentially leading to confusion. While both reference the annulus, they produce markedly different results. The clinically preferred 2D CT Distance Method measures the linear distance from the sinus midpoint to the opposite commissure. In contrast, the 3D geometric method fits a virtual circle through the widest points.

Interestingly, in our study, sheep exhibited significantly flatter sinuses than pigs only under *in vivo* conditions, suggesting a relatively higher sinus compliance in pigs (34, 35)—an observation that may reflect regional histological differences rather than contradict reports of higher ascending aortic compliance in sheep (36). This finding further increases the likelihood of compromised coronary perfusion during TAVR in the ovine model. Since the additional inflow space provided by the SOV ends at the level of the STJ—and many transcatheter devices extend beyond this point—caution is warranted when extrapolating the coronary risk observed in sheep directly to clinical scenarios. This is particularly relevant given that the STJ was found to lie 3–4 mm lower in sheep compared to pigs and humans, further limiting the available clearance for coronary inflow in the ovine model.

In addition to comparing anatomical differences between animal models, we also examined human root anatomy—both in healthy individuals and in patients with AS or regurgitation—to contextualise species-specific findings within the clinical spectrum of valve disease. In the early decades of transcatheter aortic valve development, the field was driven almost exclusively by treating AS. This focus placed priority on achieving secure device anchoring in the calcium masses without paravalvular leaks, while the baseline anatomy of healthy or regurgitant aortic roots remained largely uncharacterized. The emergence of dedicated devices for aortic regurgitation (37–39)—though developed largely through empirical approaches—has since broadened the scope of transcatheter interventions and highlighted the need for more nuanced anatomical data.

Our study provides the first direct comparison of root anatomy across healthy individuals and patients with either AS or AR. As expected, annular dimensions were smaller in AS and larger in regurgitation compared to healthy controls. However, valve pathology had no significant influence on coronary height, the lateral displacement (or “eccentricity”) of the coronary ostia from the sinus midline, or STJ height. The most pronounced anatomical difference was observed in the SOV, which was significantly narrower in AS and only modestly larger in regurgitation.

**In conclusion**, this study offers the first comprehensive anatomical comparison of the aortic root across large animal models commonly used in heart valve research and the human anatomy, both in health and in conditions typically treated by transcatheter interventions. The heterogeneity of our dataset mirrors the real-world diversity seen in preclinical programs, which often combine long-term sheep studies with short-term pig data and vary in breed and husbandry practices.

Among our most important findings were the unexpectedly “occlusion-prone” positioning of the coronary ostia in both pigs and sheep; the strong weight-dependence of annular size and LCO height in animals, independent of age; and the surprising consistency of human coronary height across valve pathologies.

These insights argue for a more tailored preclinical sizing strategy. First, weight-based animal preselection combined with transthoracic echocardiographic screening can partially mitigate the substantial anatomical variability encountered even within narrow weight brackets. However, given that even transesophageal echocardiography alone would have led to incorrect TAVR sizing in approximately half of the patients (40), our validation of *ex vivo* silicone casts against CT scans not only enables broader anatomical comparison but also reinforces the indispensable role of *in vivo* CT for accurate dimensional prediction.

Ultimately, by quantifying the extent to which species-specific anatomy—particularly in sheep—amplifies the risk of coronary obstruction, we contribute to a clearer distinction between device-related and model-related risks in valve development.

## Data Availability

The original contributions presented in the study are included in the article/Supplementary Material, further inquiries can be directed to the corresponding author.
